# Supplementary material for: Safety and data quality of EEG recorded simultaneously with multi-band fMRI
Source: PLoS One. 2021 Jul 2;16(7):e0238485. doi: 10.1371/journal.pone.0238485 (PMC8253410; doi:10.1371/journal.pone.0238485)
Supplement: Attachment — Submitted filename: EEG_fMRI_Heating_Safety_Resubmission_RevComments.docx [file pone.0238485.s002.docx]

**We thank the reviewers for their efforts and believe that their feedback has considerably strengthened the manuscript. Main text sections revised to directly address the reviewers’ suggestions are tracked in blue.**

Reviewer #1: The study investigates the temperature increase at an EEG electrode during simultaneous EEG and fMRI studies. It gives rise to several comments.

Major
The main concern is the steady and rather marked temperature increase during rest (no scanning) periods seen in figure 4, which remains unexplained. It would indeed be relevant to have the setup with temperature measurement performed without scanning a subject. However, results in the phantom (watermelon) are not reported.

**We are sorry if the manuscript wording/layout has caused confusion. We would like to clarify that *all* of the heating measurements in this study were obtained on (watermelon) phantoms, and all of our phantom measurements are reported. We have revised the manuscript to make this more clear. Most notably, we have reorganized the methods section and added respective subheadings. We have also added “phantom” and “humans” to the respective figure titles.**

**It is true that there were temperature increases during the rest period of TP7 in the phantom. However, we believe this was not due to room temperature change, because data from ECG, which were measured simultaneously showed temperature decreases during the same period. In this draft we provide data from two additional new experiments. These data show that during the rest periods, the temperature tends to drift, and the sign of this drift can be positive or negative. We have noted in the revised text that these are part of the experimental noise associated with the measurements, and may be due to local convective air currents (Page 20). The ubiquitous presence of drift underscores the importance of our experimental design, in which the different MR sequences that we compare to each other are interleaved to compensate for such effects. The interleaved design is an important aspect of our work. We have revised the text to make this point more clear (Page 19). All of the heating measurements were obtained on phantom measurements, and all of our phantom measurements are reported. We have also revised the manuscript to make this more clear.**

The multi-band technique has lower SAR than the regular EPI technique, which is an unusual design choice and doubles B0-related slice distortions and chemical shift displacement artifacts. This is not typical for multi-band methodology. As such, studying the RF safety of the multi-band technique is inconsequential.

**We agree with the reviewer that, given our parameters (multiband RF pulse duration of 5300 µs), our multi-band sequence has lower SAR than the standard single band protocol used for comparison.  This is by design, as we are aiming at a multiband pulse that is safe for simultaneous EEG and fMRI scans. The lengthening of the RF pulse is to accommodate high multiband factors. This length of RF pulse will not significantly degrade the slice profile, or create distortions that are worse than those that are routinely observed in the phase encoding direction. Additionally, the calculations and comparisons done in the paper are still valid and enable safe simultaneous acquisition. Matching duration of the standard EPI and the multiband approach would result in higher SAR for the multiband approach, which would have counter-acted the point of the paper, to keep our multiband SAR below that of the standard, single band approach.**

The description compensating the increased SAR at short TR by reducing the flip angle is misleading. The flip angle needs to be reduced to minimize signal saturation at short TR, which is customary for fast fMRI methods. Please clarify the Ernst angle at 3 T and TR: 440 ms.

**We have revised our justification for the selection of the flip angle to state the Ernst angle, and to state that we chose our flip angle so as to stay below the Ernst angle (Page 8). Given the importance of safety, the high sensitivity of SAR on the flip angle (flip angle squared), the uncertainty of the optimal or “true” Ernst angle due to T1 uncertainty, and the acceptability of data acquired below the Ernst angle (Gonzalez-Castillo et al., NeuroImage 54 (2011) 2764–2778), we believe it is sound practice to consider SAR when choosing a flip angle.**

Neither slice thickness nor readout bandwidth nor pixel size of the two fMRI methods are matched. A single subject study to compare the sensitivity of two non-matched fMRI methods is not acceptable. Please clarify the reasons for considerably higher sensitivity in the case of MB-EPI. Was a correction for autocorrelations applied?

**We would like to emphasize that the single-subject investigation into BOLD sensitivity was only a confirmatory and supplementary report. The goal of the current manuscript is not to establish functional sensitivity of MB sequences, which has been done previously and confirms higher sensitivity (e.g. Demetriou et al. 2018, doi:10.1016/j.neuroimage.2018.05.011, Todd et al. 2016 doi:10.1016/j.neuroimage.2015.08.056) likely due to better temporal resolution. To clarify that this complimentary single-subject report is not part of the investigated research question (namely safety and quality of EEG recordings), we have move it into a separate supplementary materials document. In the supplementary materials, we have added that serial autocorrelations were corrected using a FAST model for the MB sequence and an autoregressive AR(1) model for the SB-COV sequence.**

**We agree with the reviewer that bandwidth and pixel size were different between scans. The single band sequence in the main manuscript corresponds to the highest-SAR sequence recommended by the manufacturer of the EEG equipment. Therefore, it provided the comparison sequence that should not be altered. However, the temporal and spatial resolution of this sequence is poor by contemporary standards. The MB sequence allows better resolution without higher SAR. Importantly, despite the differences between protocols, the relative heating from these two different sequences, is governed simply by their relative values of RF deposition, or B1rms-max. This result is important because it indicates generality across many types of sequences and protocols. This is a central point of the paper, and we have modified the paper to make this point more clear (see the second paragraph of the Discussion, Pages 19-20). The bandwidths of the two sequences differed (2056 Hz/pixel for the MB and 2470 Hz/pixel for the SB), however heating is not expected to be sensitive to bandwidth.**

Please clarify how B1+ was measured and provide B1+ maps.

**We did not perform measurements of B1+ maps. On page 9 (line 193) we have now clarified: “B1+rms is a property of the sequence and does not depend on the properties of the object being scanned”.**

Reporting temperature for a single scalp electrode is inadequate. At least 4 temperature measurements should be performed at different locations of the scalp that are exposed to high B1+ levels.

**Thank you for motivating us to extend the dataset with new recordings. Our equipment includes only two channels. Therefore, in this draft we are providing data from two additional phantom heating tests, allowing us to sample temperature changes from two additional scalp electrodes, providing measurements from a total of four electrodes across the scalp.**

There is no comparison of EEG data quality between EPI and MB-EPI.

**We agree this is a valid concern and have recorded new data to directly address this issue. We have added data from three additional human subjects that directly compares EEG data quality between the MB sequence, the SB sequence, and recordings with no active scanning (Fig. 6).**

Minor
B1+ is not defined.

**We have updated the manuscript to state that “B1+ refers to the magnitude of the (positively rotating component of the) oscillating magnetic field generated by the RF coil” (Page 5).**

TP7 is a temporal lobe electrode, but this is not stated. The paper should be readable for a non EEG expert. Monitoring temperature at the ECG electrode is useful. However, placement and possibilities for heat exchange should be described.

**We have revised the manuscript to describe where each of the electrodes are placed during the heating experiments (Page 12). We have described the placement of the ECG electrodes in some detail in the following sentence: “Given the small size of the watermelon, the ECG electrode was routed underneath the watermelon once and placed in between the HEOG and IOG electrodes to ensure that no loop was created within the magnet (Fig 1).” We believe the figure will be helpful for the reader to understand the placement. We have updated the manuscript to include this sentence regarding heat transfer: “The monitored electrodes were exposed to the ambient air, without cushioning or other barriers to heat transfer.”** **(Page 11)**

A more detailed explanation is needed regarding the steady temperature rise seen in the rest (no scanning) periods in Fig.4. Is the room temperature changing? What are the possibilities for air exchange around the electrodes – is there a free space?

**As described above, our new data show that the temperature drifts can be positive or negative. The drift is a local effect, because it is not in the same direction at different electrodes during simultaneous measurements. This is now discussed in the manuscript. The revised manuscript also states that there is free space around the electrodes.**

Fig 3 and 4: The blue and black lines are to close and difficult to distinguish.

**We have changed the plot to maximize distinguishability.**

Please clarify what MB-EPI sequence was used.

**We have more clearly labeled the MB sequence parameter section in the methods.**

“Resolution” is the wrong description for the image matrix size.

**We have corrected this.**

Please clarify why an acquisition pixel size much smaller than the smoothing kernel width was used for this study.

**Smoothing with a kernel considerably larger than the acquired voxel size is the most common practice in cognitive neuroscience studies using fMRI. This practice is believed to increase functional signal-to-noise ratio (based on the common observation that task-related BOLD signal changes are shared more strongly among neighboring voxels than is the case for noise).**

**Please note that as stated above, the functional localizer experiment (for which smoothing was used) is only supplementary to the core goal of this paper. Accordingly, we have moved the functional fMRI section (previously Figure 1) to our supplementary materials to prevent confusion.**

Reviewer #2: The research is well planned and executed. The paper is well written, and the results are clearly presented. I have three things I wish the authors would elaborate on

1. I agree that ECG electrode is the one you definitively should measure in the heating experiment. However, in my opinion the reference 55 does not support the statement on line 271-272
“One of the probes was placed beneath the ECG electrode, because this electrode has the longest leads and is therefore expected to exhibit greater heating [55].”
Is the reference correct? The article by Angelone does not mention anything about the lead length and its impact on heating. Could you point it out, just in case I missed it. Does other research or guidelines of EEG equipment manufactured support the choice of measuring the heating of TP7?

**The reviewer is correct; the Angelone article is not a good fit. We have replaced the reference with one that does address the effect of lead length (Dempsey MF, Condon B. Thermal injuries associated with MRI. Clin Radiol. 2001;56: 457–465. doi:10.1053/crad.2000.0688 ). We have changed the justification of using TP7 to state (Page 12) that it is easily accessible, and distanced from the other scalp electrodes that have been added to the revised paper.**

2. Is the number of measured electrodes sufficient or not in the heating experiment? Are there enough repetitions? Can the results from two electrodes be generalize to all the electrodes? Mainly hoping for some discussion about the limitations of the study.

**Based on these questions we decided to collect additional data, expanding from one heating experiment to three (now covering four electrodes: ECG, TP7, TP8, ad FP2). This expansion allows us to better quantify the heating differences between the sequences, and to demonstrate a statistically significant difference in the heating between the sequences (Page 15). We have also revised the text to include a discussion of how measurement noise limits our ability to draw conclusions about any differences in the shapes of the heating spatial distributions between the two sequences (Page 20).**

3. I wish that there would have been some statistical analysis of the heating rate and ratio (standard deviation). Or is there a reason for not doing it? Is there a significant difference between the measured and the theoretical value?

**We have updated the paper with standard deviation values of the heating ratios (Table 1). The standard deviations are large, and the theoretically expected heating ratio is well within this range. The paper has been updated to state this. As mentioned above, we are now also reporting a statistical evaluation of the difference in mean heating between the MB and non-MB sequences.**

Minor things:

There are discrepancies in the text
-In the use of hyphen. In the beginning is used e.g. “MB-fMRI”, and in the abstract and latter part of the paper is used “MB fMRI”. I do not know which is grammatically correct.
- use of space before the physical quantity is sometimes missing e.g. 3000ms vs. 3000 ms. As far as I know, there should be a space between the number and the quantity.
-Decide which format to use in the text there are TR=3000, TR: 1900 ms. This even. varies within a sentence e.g. “0.9 mm isotropic, TR: 1900 ms, TI: 900 ms, TE = 2.32 ms, GRAPPA factor = 2) (line 155-156).

**These have been corrected.**

Line 51 SNR (declared later in the text on line 59)

**This has been corrected.**

Line 91 Maybe the abbreviation SAR should be within parenthesis (SAR).

**This has been corrected.**

Line 73: Maybe a typo in the number “including 2,250ms silence.”? Elsewhere numbers >= 1000 have been written without a comma.

**This has been corrected.**

Line 181 MNI space - reference missing?

**A reference has been added.**

Few abbreviations that have not been declared or assumed as common knowledge. Line 256 FIR and on line 263 ICA

**This has been corrected.**

Reviewer #3: The authors present a study where they first run a pilot MB protocol with a functional localizer task to evaluate MB-fMRI data quality (Figure 1). They then proceed to demonstrate the safety of simultaneous EEG/MB-fMRI with a capped watermelon with thermometers attached (Figures 2-4). Following from this, 6 human participants were recruited to be scanned for a total of 20 minutes resting-state eyes closed each (Figures 5-6).

It is not immediately clear which “larger experiment” (refer to line 211) the authors are citing.

**We are sorry for the confusion that the notion of “larger experiment” has caused. We were referring to the fact that the presented data was collected within the context of a cognitive neuroscience experiment. For that “larger” task-based experiment, we first sought to establish that EEG is safe and of good quality when recorded concurrently with the chosen MB sequence. Therefore, the present study needed to be completed first. Since there is no need for the reader to know about this “larger” context, we have removed that wording.**

There are several concerns that should be addressed:

Major Corrections

1. The authors claim that the MB sequence can be considered safe because the measured heating was below that of the “safe” SB-RF sequence (lines 296/297). As shown in Figure 4 (TP7), there was a continuous temperature rise – even during the “black line” periods (of no scanning). Were there any room temperature measurements – to potentially account for the continuous temperature rise?

**It is true that there were temperature increases during the rest periods of TP7. However, we believe this was not due to room temperature change, because data from ECG, which were measured simultaneously showed temperature decreases during the same period. In this draft we provide data from two newly recorded additional phantom heating experiments. These data show that during the rest periods, the temperature tends to drift, and the sign of this drift can be positive or negative. We have noted in the revised text that these are part of the experimental noise associated with the measurements and may be due to local convective air currents (Page 20).**

2. The authors claim that the EEG data recorded is of acceptable quality (Line 35). However, the authors do not compare the EEG spectra to any ‘gold standard’ paradigm – as the authors rightfully say in lines 410-411, they could compare the EEG data quality between MB, SB, and scanner-off recordings. To properly justify their claim of acceptable EEG data quality, some more rigorous comparisons to an appropriate control would be needed.

**We thank the reviewer for this suggestion, and have recorded new data from three additional subjects that directly compares EEG data quality between the MB sequence, the SB sequence, and recordings with no active scanning. For all subjects the EEG recorded during the MB sequence showed a similarly clean power spectrum compared to the SB sequence, and both were comparable to the scanner-off condition (new Fig. 6, and results section on Page 18).**

3. Regarding thermal safety whilst scanning humans; it is somewhat surprising that thermal measurements were not conducted simultaneously. If they were conducted, they should definitely be presented to further illustrate the practical safety of simultaneous EEG/MB-fMRI – as participant effects (e.g. physiological mechanisms to maintain temperature) are not reproducible on a watermelon. If they were not conducted, perhaps the authors could also include whether any individuals felt any heating sensations or if any heating related incidents occurred – to further give reassurance of the safety of simultaneous EEG/MB-fMRI.

**We agree that human data would be useful. However human measurements were not part of our original study design. Our approach was to quantify the relative heating of the MB sequence compared to a standard SB sequence, and to demonstrate that the heating ratio depends on time-averaged RF power. This heating ratio should be the same for both phantoms and humans, and we have modified the paper to make this point. We have also modified the paper to state that “…our MB sequence has now been used in over 100 scan sessions at our local imaging center, without reports of heating sensations or burns from the subjects” (Page 15).**

4. The present study does not really assess the data quality of the multiband fMRI data (whilst simultaneously recording EEG) – it only assesses the EEG data quality. Figure 1 reflects that of a pilot person of n=1 comparing between multiband and single fMRI scans – which appears fine. However, the authors could potentially go further to compare between the multiband fMRI and single band fMRI resting state scans they conducted with their n=6 main study – to see if there were any differences due to the different scan parameters. (For example, characterisation of resting state networks). This would justify the implication of the manuscript title – that the data quality of fMRI was also evaluated (during simultaneous acquisition of EEG/fMRI).

**We agree with the reviewer - the focus of our paper is on the EEG quality (and safety) obtained during the scan, and a comparison of the fMRI data quality was not the intention. We have updated the title of the paper to better reflect this and removed the implication of fMRI comparisons. We have also moved the functional fMRI section (previously Figure 1) to our Supplementary Materials to prevent confusion. On Page 9 we make explicit that good functional SNR of MB fMRI has been extensively demonstrated elsewhere and is not within our scope: “Of note, although not part of the research goal of the current investigation, we include a complimentary report of a task-based functional localizer using the MB sequence in a single human subject (Supplementary Materials). In line with extensive prior literature [16,17], we observed good functional sensitivity with this sequence. “**

Minor Editorial Comments
- In Figure 3 and 4, consider capitalising the x-axis title “Time”, and including the “degrees” symbol in the y-axis title for ˚C

**These have been modified.**

- In Table 1, perhaps the table caption could also include the word “average” – to indicate that it is the comparison of “average” heating.

**This has been updated accordingly.**

- The phrase including the word “sandbagged” in Line 220, whilst understandable in this context, could nonetheless be rephrased to avoid any malicious connotation. Consider changing to: “…were strapped down with sandbags to a stabilizing…”

**We have modified this to avoid the term.**
