# Supplementary material for: Safety and data quality of EEG recorded simultaneously with multi-band fMRI
Source: PLoS One. 2021 Jul 2;16(7):e0238485. doi: 10.1371/journal.pone.0238485 (PMC8253410; doi:10.1371/journal.pone.0238485)
Supplement: Attachment — Submitted filename: EEGSafety_ReSub2_ReviewerComments.docx [file pone.0238485.s003.docx]

**In response to Question 3 in the ‘Comments to the Author’**

3. Has the statistical analysis been performed appropriately and rigorously?

Reviewer #1: N/A

**Please note that paired-sample statistics are included for comparison of heating across the MB sequence of interest and the “control” SB sequence.**

--------------------------------

**In response to the ‘Review Comments to the Author’**

Reviewer #1: The authors have addressed out questions and criticism. However, the study still gives rise to concern.
 Major concerns

- Temperature measurements in a watermelon are not representative of in vivo conditions. The lack of in vivo temperature measurements greatly diminishes the impact of the study.

**We agree that the paper would have been stronger with in vivo temperature measurements. However, temperature measurements of phantoms are ubiquitous in the safety literature and have proven very informative for measuring heating in the context of concurrent EEG-fMRI in prior literature both with (e.g. Foged et al, Chen et al, Kuusela et al (2015)) but also without additional in vivo recordings (e.g. Uji et al).**

**Phantoms are less useful for absolute heating measurements since absolute measures may indeed differ from those in humans. However, the *difference* in heating across sequences (i.e. relative heating measurements, which are the focus of our paper) is less likely to be affected substantially by the imaged object. Importantly, our results demonstrate that temperature measurements in phantoms are suitable for statistical comparison across different sequences, i.e. of *relative* heating. Our work thus not only demonstrates the safety of a single MB sequence, but has impact in terms of demonstrating relative measurements as a practical approach that other investigators can adopt to test their own specific sequences.**

- The temperature drifts with and without EPI and MB-EPI make interpretation of safety related temperature increases very difficult. In a phantom these drifts should be controllable.

**Variance is common for all temperature measurements. Importantly, the statistical analysis provided inherently accounts for this variance. Please also note that direct control of temperature drift would not be representative of human scan conditions.**

- The authors use a downgraded MB-EPI sequence with possibly distorted slice profiles and increased chemical shift displacement in the slice direction They do not characterize it in phantoms and in vivo: "We would like to emphasize that the single-subject investigation into BOLD sensitivity was only a confirmatory and supplementary report.”.

**As we already included in our previous response letter, the “length of RF pulse used in our MB sequence will not significantly degrade the slice profile.” It is true that longer pulse durations lead to greater slice distortion and increased chemical shift displacement. However, our pulse duration of 5300 us is comparable to other protocols that have resulted in high-quality fMRI data enabling a very large set of informative neuroscience investigations. For example, the HCP VE11C Lifespan development and HCP aging protocol uses a pulse duration of 6600 us:**

<https://www.humanconnectome.org/study/hcp-lifespan-development/project-protocol/imaging-protocols-hcp-development>

**and the Nathan Kline Institute Rockland Sample features a multi-band protocol with a pulse duration of 5120 us:**

<http://fcon_1000.projects.nitrc.org/indi/enhanced/mri_protocol.html>

**The Human Connectome Project (HCP) Young Adults dataset uses a longer pulse duration of 7120 us:**

<https://www.humanconnectome.org/storage/app/media/documentation/s1200/HCP_S1200_Release_Appendix_I.pdf>

**and has resulted in a wealth of research publications:**

<https://www.humanconnectome.org/study/hcp-young-adult/publications>**.**

**Please note that we did not choose this MB sequence for the sole purposes of finding an arbitrary sequence that is safe. We chose this sequence because it meets numerous features that we needed for our cognitive neuroscience research. Specifically, we wanted high temporal resolution (desired to be below =<0.5 sec TR; achieved: 440ms) combined with whole-cerebrum coverage (desired to be >10cm; achieved: 10.78cm) at good spatial resolution (desired ~3x3x3mm; achieved: 1.8x1.8x3.5mm) while not exceeding a MB factor of 4. Once we found the ideal sequence combining these features, we then aimed at demonstrating its safety. To achieve the latter, we made sure that the sequence has relatively low SAR, which as we discussed above did not involve sacrificing data quality to a substantial degree (meaning levels of distortion that would affect the ability to answer neuroscientific questions). Given the combination of spatial and temporal resolution with safety, we believe that this sequence will actually prove very useful for the cognitive neuroscience community.**

**Further, as noted above our work will also have impact in terms of demonstrating relative measurements as a practical approach that other investigators can adopt to test their own specific sequences.**

- What’s the point if the authors already know that MB-EPI SAR will be lower?: "Matching duration of the standard EPI and the multiband approach would result in higher SAR for the multiband approach, which would have counter-acted the point of the paper, to keep our multiband SAR below that of the standard, single band approach.”

**It is true that the assumption that heating is proportional to power deposition across all sequences is reasonable. However, given the importance of safety, the research community, and the manufacturer of the EEG equipment, have proceeded cautiously, and reacquired additional evidence. Our results add to this evidence and confirm that the assumption is valid in the transition to MB sequences.**

There are problems with the meaning of the MR physics: “This length of RF pulse will not significantly degrade the slice profile, or create distortions that are worse than those that are routinely observed in the phase encoding direction….. B1+rms is a property of the sequence and does not depend on the properties of the object being scanned”.

**We believe that the statement “This length of RF pulse will not significantly degrade the slice profile” is well supported by our above-described considerations in response to the reviewer’s question on slice distortions. We agree with the reviewer that a comparison of slice distortions with the typically much larger distortions routinely observed in the phase encoding direction is not directly relevant to the discussion (though not incorrect). Our intention was simply to point out that significant distortions do exist in fMRI and are usually part of tradeoffs made for imaging. Please note that this comparison is not part of the manuscript.**

**The statement that “B1+rms is a property of the sequence and does not depend on the properties of the object being scanned” appears in the paper and is a true statement. The B1+ field scales with the flip angle divided by the pulse duration and the gyromagnetic ratio. The flip angle and the pulse duration are properties of the sequence that do not depend on the properties of the object being scanned, and the gyromagnetic ratio is a physical constant.**

The statement by the authors: "We agree with the reviewer that bandwidth and pixel size were different between scans…...However, the ... spatial resolution of this sequence is poor by contemporary standards" does not clarify and adjust the question regarding pixel size and readout bandwidth.

**We are sorry but it is not clear to us what is the question regarding pixel size and bandwidth. The reviewer’s original comment was, “Neither slice thickness nor readout bandwidth nor pixel size of the two fMRI methods are matched. A single subject study to compare the sensitivity of two non-matched fMRI methods is not acceptable. Please clarify the reasons for considerably higher sensitivity in the case of MB-EPI.” In this comment, the reviewer requested that we clarify the reasons for the higher sensitivity of the MB-EPI. In our response, we provided these reasons. Does the reviewer have concerns about our justification? If so, we would appreciate it if the reviewer explained them.**

**Please also let us reiterate that our current study does not aim at investigating or comparing the quality of the fMRI sequences; we have answered above why we do not believe such demonstration would be necessary given the established quality of MB sequences.**

EEG power spectral density above about 20 Hz decreases when scanning. I am not sure whether that explains it: "a likely side effect of GA and BCG artifact subtraction”.

**We agree that it is difficult to specifically pinpoint the origin of this signal loss. However, the observation that the EEG gamma band is particularly heavily affected by gradient artifacts (and consequently GA removal) is well-known in the EEG-fMRI literature. We have extended the discussion regarding this effect (p. 18) “In particular, because GA causes stronger contamination in the high EEG frequencies (>~20Hz, cf. Fig 4), the brain signal that can be recovered from this frequency range through GA removal is lower than in slower EEG frequencies (Ryali et al., 2009). Note that the loss of high-frequency EEG signals observed for the MB sequence were closely aligned with those observed in the SB sequence (Fig. 6).”.**

Reviewer #2: The authors have addressed all comments. In results section is presented some statistical analysis (paired t-test), but this is not presented in the Discussion part. However, I don't think this is an issue.

Reviewer #3: The authors have since included more phantom controls along with human subject controls, which much more appropriately justify the authors’ conclusions. This reviewer is largely satisfied with the edits; but have included several minor suggestions.

Suggestions
 - Line 250: “…this electrode has the longest leads and is therefore…” Consider changing to: "...this electrode has the longest lead and is therefore..."

**Thank you, this is changed.**

- Line 265-266: “The first pair of MB/SB runs was discarded from experiment 2 due to insufficient equilibration of the temperature probe with ambient temperature at the beginning of the scanning session.” How was “sufficient equilibration” determined? Perhaps a sentence to describe what exactly happened which led to the omission of data would be appropriate.

**We have revised the text to add more detail (p. 13):**

**The original text read:**

**“The first pair of MB /SB runs was discarded from experiment 2 due to insufficient equilibration of the temperature probe with ambient temperature at the beginning of the scanning session.”**

**The revised text reads:**

**“The first pair of MB /SB runs was discarded from experiment 2 because scanning commenced before the phantom had equilibrated with room temperature. This was apparent from the fact that during the first run, and the rest periods before and after, the temperature of the probes decreased as the watermelon equilibrated with the colder environment of the scan room.”**

- Line 277: “fMRI-free” – please detail: was the EEG conducted on the person inside or outside the MRI scanner room? The reason why I ask is that this will determine whether the BCG artefact is still markedly present or not – and therefore improve replicability for future investigations.

**Thank you for drawing our attention to this omission. We have clarified the following line to: “The three subjects underwent 5 minutes of data acquisition inside the MRI scanner for each of the MB, fMRI-free, and SB conditions, in that order.” (p. 13). Additionally, the reviewer’s comment made us realize that we had provided Figure 6 without using the BCG-corrected EEG data for the fMRI-free condition. The newly uploaded Figure 6 has the proper BCG-corrected fMRI-free data in the graph.**

- Line 323-325: The current problem with simultaneous EEG/MB-fMRI is that there is a heating concern when conducting simultaneous EEG/MB-fMRI – not when MB-fMRI is solely conducted without an EEG cap on the human participant. Have over 100 scan sessions with simultaneous EEG/MB-fMRI been performed already? (I assume not). I appreciate that the goal was to demonstrate the theoretical link between heating ratio and time-averaged RF power; but the practical safety of simultaneous EEG/MB-fMRI on human participants should still be assessed to absolutely reinforce the safety of simultaneous EEG/MB-fMRI. I assume that temperature readings were not conducted simultaneously with simultaneous EEG/MB-fMRI on the human participants (since they have not been included). However, instead of temperature readings, perhaps a comment could be made regarding the three human participants (as shown in Figure 6) who received simultaneous EEG/SB-fMRI, fMRI-free, and simultaneous EEG/MB-fMRI; and whether these three humans reported any differing heating sensations with the different scans. Whilst it is great that the safety is determined on watermelon phantoms, it is of pertinent importance that safety is determined on human participants.

**We are sorry that we were not clear about the fact that 2/3 of the >100 human scan sessions with this MB sequence were indeed recorded with concurrent EEG. We have revised the respective sentence accordingly (p. 22) “Although temperature measurements were not performed on humans during scanning, our MB sequence has now been used with concurrent EEG in over 70 scan sessions at our local imaging center, without reports of heating sensations or burns from the subjects.”**

**Per the reviewer’s suggestion, we have further added (p.22): “Further, the three subjects that underwent simultaneous EEG-SB-fMRI and fMRI-free EEG in addition to EEG-MB-fMRI (cf. Fig. 6 for EEG data) did not report any heating sensation in any of the conditions.”**
